# Health websites on COVID-19: are they readable and credible enough to help public self-care?

**DOI:** 10.5195/jmla.2021.1020

**Published:** 2021-01-01

**Authors:** Saeideh Valizadeh-Haghi, Yasser Khazaal, Shahabedin Rahmatizadeh

**Affiliations:** 1 saeideh.valizadeh@gmail.com, Department of Medical Library and Information Sciences, School of Allied Medical Sciences, Shahid Beheshti University of Medical Sciences, Tehran, Iran; 2 yasser.khazaal@chuv.ch, Department of Psychiatry, Lausanne University Hospitals and Lausanne University, Lausanne, Switzerland; 3 shahab.rahmatizadeh@gmail.com, Department of Health Information Technology and Management, School of Allied Medical Sciences, Shahid Beheshti University of Medical Sciences, Tehran, Iran

## Abstract

**Objective:**

There are concerns about nonscientific and/or unclear information on the coronavirus disease 2019 (COVID-19) that is available on the Internet. Furthermore, people's ability to understand health information varies and depends on their skills in reading and interpreting information. This study aims to evaluate the readability and creditability of websites with COVID-19-related information.

**Methods:**

The search terms “coronavirus,” “COVID,” and “COVID-19” were input into Google. The websites of the first thirty results for each search term were evaluated in terms of their credibility and readability using the Health On the Net Foundation code of conduct (HONcode) and Flesch-Kincaid Grade Level (FKGL), Simple Measure of Gobbledygook (SMOG), Gunning Fog, and Flesch Reading Ease Score (FRE) scales, respectively.

**Results:**

The readability of COVID-19-related health information on websites was suitable for high school graduates or college students and, thus, was far above the recommended readability level. Most websites that were examined (87.2%) had not been officially certified by HONcode. There was no significant difference in the readability scores of websites with and without HONcode certification.

**Conclusion:**

These results suggest that organizations should improve the readability of their websites and provide information that more people can understand. This could lead to greater health literacy, less health anxiety, and the provision of better preventive information about the disease.

**Figure:**
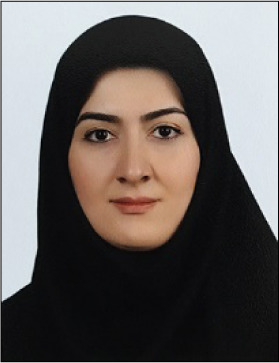
Saeideh Valizadeh-Haghi

## INTRODUCTION

As a large family of viruses, coronaviruses are responsible for multiple diseases. In humans, these include the common cold alongside severe diseases such as Middle East respiratory syndrome (MERS-CoV) and severe acute respiratory syndrome (SARS-CoV) [1]. Coronavirus disease 2019 (COVID-19) was first reported in Wuhan, China, on December 31, 2019 [2]. According to a report published by the World Health Organization (WHO) in May 2020, COVID-19 spread across 216 countries very rapidly [3]. As a vaccine for this virus has yet to be developed, non-pharmacological interventions, such as increased hygiene, are crucial for controlling the virus and reducing the risk of infection [4, 5].

Many people consider the Internet to be a useful and important source of health information [6–9] that can encourage the use of preventive strategies and consultation with physicians [10]. On March 13, 2020, Google Trends reported that the term “coronavirus” was searched five million times (supplemental appendix), underlining the importance of the Internet as a source for health information. However, multiple studies demonstrate that health websites may not be credible and may contain inaccurate or misleading information [11–16]. Therefore, the information found on health websites could increase uncertainty and anxiety and, thereby, serve to harm people's health [17–19].

Trust in online health information has recently been of great concern [20] due to deficiencies in people's ability to judge the quality of this information [21, 22]. The current COVID-19 pandemic further raises concerns about nonscientific, misleading, and unclear information disseminated via the Internet or other media [23, 24], which can increase anxiety among the population. This pandemic may be particularly problematic for people with cyberchondria, defined as compulsive searching for health information online [25], because the existence of inaccurate information on the Internet could serve to increase this behavior. Apart from individuals with health anxiety, the pandemic's high mortality rate causes widespread fear, anxiety, and stress for people around the globe [26].

Although different motivations can contribute to online health information-seeking, searching for information about COVID-19 online can be understood as one way to cope with the stress associated with the pandemic [27]. For instance, people may search for information about COVID-19 to gain clarity about their own or a relative's symptoms as an attempt to alleviate uncertainty [28]. However, the fear and anxiety caused by pandemics such as COVID-19 can serve as an additional barrier to understanding information correctly [29] and may lead to further anxiety, inappropriate use of information, and unnecessary referrals to medical centers.

The proper use of health information depends on people's ability to understand, interpret, and comprehend that information [30] and can be influenced by many factors, including its readability [31]. To be considered readable, a text should be easy to read and contain concepts that are easy to understand [32]. Organizations such as the American Medical Association (AMA) and National Institutes of Health (NIH) advise that the readability of health information should not exceed a sixth-grade level [33] and should be understandable to eleven years olds [34]. Moreover, it should not use medical jargon [35]. However, online health information is often written in at a level that is not easily understood by many people [36–41].

Considering the increased use of the Internet to obtain health information on COVID-19 [42], it is of great importance to understand the readability and credibility of health websites. The objective of this study was to evaluate the readability and credibility of publicly accessible health websites containing COVID-19-related information.

## METHODS

### Website selection and categorization

The authors searched for “coronavirus,” “COVID,” and “COVID-19” using Google, the most frequently used Internet search engine [43, 44], on February 26, 2020. The search was performed using Google Chrome, and the browser history, cached images, and cookies were removed before the searches were carried out. As 90% of all search engine users browse only the first 3 pages of results pages (i.e., the first 30 results) [45], the first 30 websites returned by Google for each of the 3 keywords were examined, resulting in a total of 90 websites.

**Figure 1:**
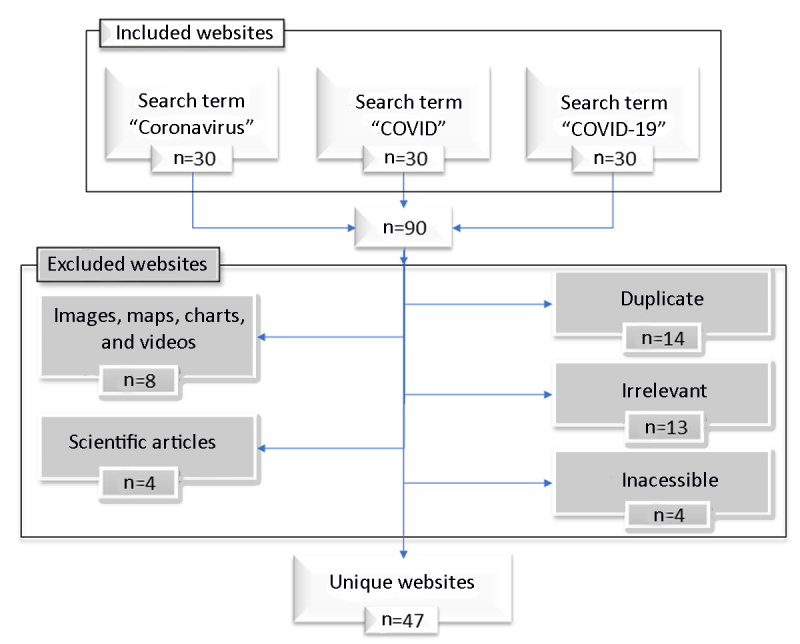
Google search flow diagram for website retrieval

We excluded non-English-language websites, irrelevant websites, scientific papers, duplicate websites, inaccessible websites, primarily non-text-based websites (e.g., YouTube), and advertisement-sponsored links. Based on these criteria, forty-three websites were excluded, and forty-seven unique websites were selected for evaluation (Figure 1). These websites were then divided into the following five categories according to their statement of affiliation: commercial, news, educational, governmental, and organizational.

### Readability evaluation tools

Four readability scales, each of which uses different techniques, were used to evaluate the readability of the websites: Flesch-Kincaid Grade Level (FKGL), Simple Measure of Gobbledygook (SMOG), Gunning Fog, and Flesch Reading Ease Score (FRE). These scales have been used in many readability evaluation studies of health websites on various topics and are reliable [46–53], and NIH recommends using FKGL, Gunning Fog, and SMOG for readability evaluations [54].

The FRE scale produces a score between 0 and 100, with higher scores indicating higher readability levels: scores of 90–100, 60–70, and 0–30 indicate that text can be understood by fifth-grade students, eighth- or ninth-grade students, and university graduates, respectively. The Gunning Fog scale produces scores of 5, 10, 15, or 20, which indicate that text is easy to read, hard to read, difficult to read, or very difficult to read, respectively. The SMOG and FKGL scales estimate the years of education a person needs to have completed to understand a written text; for example, a score of 7.4 indicates that a seventh-grader can understand the text. To apply these scales, a free online readability checker (Readability Formulas) was utilized [55]. This web-based tool has been used in readability evaluations of a wide variety of health-related websites [36, 46, 50, 56–59].

### Credibility evaluation tool

Various tools—including JAMA [60], DISCERN [61], and Health On the Net Foundation code of conduct (HONcode) [62]—are available for evaluating the credibility of health websites. The HONcode consists of nine criteria: authority, complementarity, privacy, attribution, justifiability, transparency, financial disclosure, and advertising policy. The nonprofit HON Foundation, which is officially related to WHO, checks the credibility of websites at the request of the institutions hosting the websites. If they meet the criteria, the HON logo is placed on the website, which indicates that the website has been officially certified and is a reliable source of health information. The HON Foundation also provides a toolbar extension compatible with Chrome and Firefox browsers that helps people easily identify HONcode-certified websites while browsing [63]. HONcode is the oldest and most-used ethical and trustworthiness code for medical and health-related information available on the Internet [64]. It is reliable and has been used in multiple studies to assess the credibility of health websites [11, 12, 14–16, 65–67]. Therefore, we used the HONcode toolbar to identify certified websites containing information on COVID-19.

### Statistical analysis

We tested whether HONcode-certified websites were more readable than non-HONcode-certified websites using independent *t*-tests. We also tested whether readability scores differed among website categories and depended on the website position on the search results pages using one-way analysis of variance (ANOVA). Statistical analysis was conducted using SPSS 18.

## RESULTS

We analyzed forty-seven unique websites containing information on COVID-19 retrieved via Google. The retrieved websites appearing on the first page of the search results included more HONcode-certified websites than those on the second and third pages (Table 1). However, even on the first page of results, most websites were not officially approved. In total, only six websites were HONcode-certified, and all were commercial and/or organizational.

**Table 1:** Frequency and categorization of websites retrieved via Google

Variables	Health On the Net Foundation code of conduct (HONcode) certified				Total
	Yes		No		
Search results page					
1	4	(27%)	11	(73%)	15
2	1	(7%)	14	(93%)	15
3	1	(6%)	16	(94%)	17
Category					
News	0	(—)	14	(100%)	14
Governmental	0	(—)	18	(100%)	18
Commercial	2	(40%)	3	(60%)	5
Organization	4	(44%)	5	(56%)	9
Educational	0	(—)	1	(100%)	1
Total	6	(13%)	41	(87%)	47

ANOVA showed no significant effect of website category on readability scores (Table 2). There was also no significant effect of search results page number on readability scores (Table 3).

**Table 2:** Readability scores of websites according to their category

Reada-bility formula	Mean (SD)													*p*-value
	News			Governmental			Commercial			Organizational			Educa-tional*	
FRE	51.8	(7.2)	Fairly difficult	44.5	(14.1)	Difficult	52.8	(6.0)	Fairly difficult	42.2	(13.4)	Difficult	45.5	0.212
Gunning Fog	12.9	(2.2)	Hard to read	13.4	(2.5)	Hard to read	12.2	(1.2)	Hard to read	13.9	(2.7)	Hard to read	14.2	0.687
FKGL	11.0	(2.1)	11th grade	11.6	(2.4)	11th grade	10.4	(1.5)	10th grade	11.9	(2.4)	12th grade	12.7	0.681
SMOG	9.9	(1.5)	10th grade	10.5	(2.0)	10th grade	9.4	(0.9)	9th grade	10.6	(1.9)	10th grade	11.4	0.596

* There was only one website in this category.

SD=standard deviation, FKGL=Flesch-Kincaid Grade Level, SMOG=Simple Measure of Gobbledygook, FRE=Flesch Reading Ease Score.

**Table 3:** Readability scores of websites according to their search results page number

Readability formula	Mean (SD)									*p*-value
	Page 1			Page 2			Page 3			
FRE	47.0	(14.3)	Difficult	47.7	(7.8)	Difficult	46.7	(13.1)	Difficult	0.971
Gunning Fog	13.1	(2.5)	Hard to read	13.3	(2.1)	Hard to read	13.3	(2.5)	Hard to read	0.949
FKGL	11.4	(2.4)	11th grade	11.4	(1.7)	11th grade	11.3	(2.5)	11th grade	0.992
SMOG	10.1	(2.0)	10th grade	10.3	(1.3)	10th grade	10.3	(2.0)	10th grade	0.935

*T*-tests showed no significant differences in readability scores between HONcode-certified and non-HONcode-certified websites (Table 4).

**Table 4:** Readability scores of websites depending on their Health On the Net Foundation code of conduct (HONcode) certification

Readability formula	Mean (SD)							*p*-value
	HONcode-certified			Non-HONcode-certified				
FRE	46.2	(16.7)	Difficult	47.3	(11.2)	Difficult	0.884
Gunning Fog	12.8	(3.0)	Hard to read	13.3	(2.2)	Hard to read	0.648
FKGL	11.1	(2.9)	11th grade	11.4	(2.1)	11th grade	0.725
SMOG	9.8	(2.3)	10th grade	10.3	(1.7)	10th grade	0.541

Among prominent international and national organizations, the readability of website content published by the National Health Service (NHS) and Centers for Disease Control and Prevention (CDC) scored highest and lowest, respectively (Table 5). The readability level of content available through the WHO and NIH websites was “difficult to read.”

**Table 5:** Readability levels for websites published by prominent international and national organizations

Organization	Grade level	Reading level	Users' age and grade level	
Centers for Disease Control and Prevention (CDC), https://www.cdc.gov	16	Very difficult to read	22 years old and older	College graduate
National Institutes of Health (NIH), https://www.nih.gov	13	Difficult to read	18–19 years old	College level entry
World Health Organization (WHO), https://www.who.int	12	Difficult to read	17–18 years old	12th graders
European Centre for Disease Prevention and Control (ECDC), https://www.ecdc.europa.eu/en	12	Difficult to read	17–18 years old	12th graders
GOV.UK, https://www.gov.uk	11	Fairly difficult to read	15–17 years old	10th and 11th graders
Patient, https://patient.info	10	Standard/average	14–15 years old	9th and 10th graders
Healthline, https://www.healthline.com	9	Fairly difficult to read	13–15 years old	8th and 9th graders
Australian Government Department of Health, https://www.health.gov.au	8	Standard/average	12–14 years old	7th and 8th graders
New Zealand Government Ministry of Health, https://www.health.govt.nz	8	Standard/average	12–14 years old	7th and 8th graders
National Health Service (NHS), https://www.nhs.uk	7	Fairly easy to read	11–13 years old	6th and 7th graders

## DISCUSSION

Low literacy levels are a barrier to health knowledge. One solution to this problem is to provide content in plain language that is easy to read [68]. Using plain language can help convey information to a wider population [69] and allows users to find what they need, understand what they have found, and then use this information to meet their needs [70]. On a global level, individual reading abilities and the readability levels of consumer information contribute to the overall health of all people and society [71]. Due to the importance of readability in the context of health literacy, health promotion, and patient self-care, we evaluated the readability and credibility of COVID-19 information available on websites intended for the general public that were retrieved via Google searches.

Patient education materials should be easily understood by an average eleven-year-old or students in the sixth grade [34]. The results of this study show that the readability of COVID-19 information on websites is more advanced than the recommended level and is generally aimed at high school graduates or college students. This finding is consistent with studies examining online information on Ebola [72] and other diseases [73–75]. Moreover, our findings show that the readability level of website content published by international and national health organizations such as WHO and CDC was far above the recommended sixth-grade reading level. This is concerning because individuals consider these websites to be major sources of reliable health information, especially in health crises such as the current COVID-19 outbreak.

We also examined the readability levels of websites based on their category. Government websites were expected to be more readable than other types of websites, because their purpose is usually to educate the general public [76]. However, we found that the readability scores of websites in all categories, including governmental websites, were far above the recommended level. Although previous studies reported that governmental websites were more readable than other types of websites [73], we found that the readability of commercial websites was more suitable for a public audience than governmental websites. However, it should be noted that the information on commercial websites has been found to be of lower quality than other types of websites [77, 78]. Consequently, people looking for information on the symptoms, prevention, treatment, and management of COVID-19 may come across websites that contain readable but inaccurate information or, conversely, accurate but unreadable information, meaning that decisions based on this information could increase their anxiety and even threaten their health [17–19].

Most people tend to browse search results presented on the first page [74, 79]. Therefore, we expected that websites appearing on the first page of the search results would be more readable than those on the second and third pages. However, we found no significant difference in the mean readability scores of websites appearing on different pages of the search results. While organizations make efforts to increase the rankings of their websites in search engine results, we recommend that they also pay attention to the readability of their websites to ensure that their content can be understood. This would allow users to better understand the websites' content, satisfy their information needs, and help to prevent the spread of dangerous infectious diseases such as COVID-19.

We found that most websites that we examined were not officially certified by the HON Foundation. Therefore, individuals searching for information on COVID-19 may encounter websites that contain misinformation, which could lead to incorrect decision-making and anxiety. Moreover, both HONcode-certified and non-certified websites had poor readability scores. This finding contrasts with a similar study on prostate health, in which more credible websites were found to have better readability [80]. Therefore, we recommend that authoritative organizations providing health information about various infectious diseases, including COVID-19, pay more attention to increasing the readability of their websites to help people understand the information that is provided.

### Limitations

There were some limitations in this study. For example, searches were conducted through Google Search, and using other search engines might have generated different results. In addition, due to the dynamic characteristics of websites, alternative results might have been obtained if the searches were conducted at different times.

## SUPPLEMENTAL FILE

**Appendix:** Interest in coronavirus-related searches over time, based on Google trends
