# Supplementary material for: Health websites on COVID-19: are they readable and credible enough to help public self-care?
Source: J Med Libr Assoc. 2021 Jan 1;109(1):75–83. doi: 10.5195/jmla.2021.1020 (PMC7772974; doi:10.5195/jmla.2021.1020)

Journal of the Medical Library Association

Supplemental content to
J Med Libr Assoc. Jan;109(1):dx.doi.org/10.5195/jmla.2021.1020
www.jmla.mlanet.org
© Valizadeh-Haghi, Khazaal, Rahmatizadeh 2021

Health websites on COVID-19: are they readable and credible enough to help public self-care?

Saeideh Valizadeh-Haghi, PhD; Yasser Khazaal, MD; Shahabedin Rahmatizadeh, PhD

APPENDIX

Interest in coronavirus-related searches over time, based on Google trends

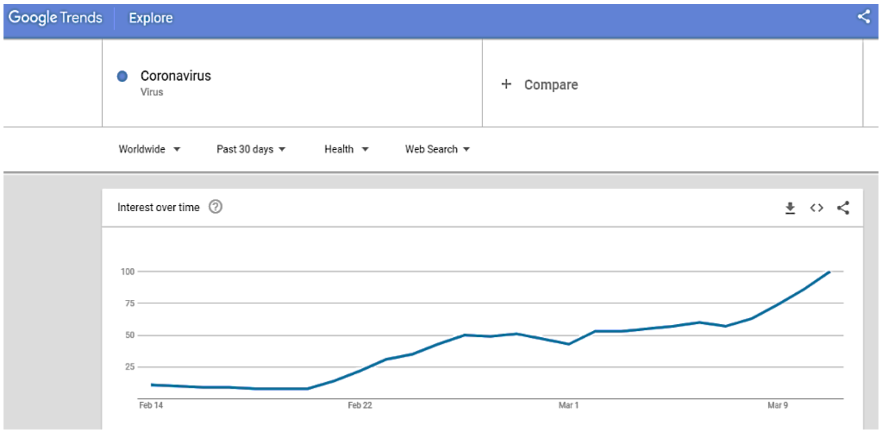

Supplement: Supplementary file 1 — Appendix: Interest in coronavirus-related searches over time, based on Google trends [file jmla-109-1-75-s01.pdf]
